# Integration of cervical cancer screening into HIV/AIDS care in low-income countries: a moral imperative

**DOI:** 10.3332/ecancer.2021.1237

**Published:** 2021-05-20

**Authors:** Chemtai Mungo, Emily Barker, Magdalene Randa, Jeniffer Ambaka, Cirilus Ogollah Osongo

**Affiliations:** 1 Division of Prevention Science, University of California, San Francisco, 550 16th St 3843, Mail Code 0886, San Francisco, CA 94143, USA; 2 Department of Obstetrics and Gynecology, Rush University School of Medicine, 600 S Paulina St, Suite 438, Chicago, IL 60612, USA; 3 Family AIDS Care and Education Services (FACES) Clinic, Lumumba Hospital, FACES Building, Agoi Road, Ondiek PO Box 614-40100, Kisumu, Kenya

**Keywords:** cervical cancer elimination, HIV and HPV, low- and middle-income countries

## Abstract

Although cervical cancer is preventable, in 2018, approximately 570,000 new cases occurred globally. Cervical cancer disproportionately affects low- and middle-income countries (LMICs), which accounted for 90% of deaths in 2018. Women living with the Human Immunodeficiency Virus (WLWH) are at increased risk of cervical cancer and are in urgent need of prevention. Despite evidence-based guidelines for screening and prevention of cervical cancer, the majority of WLWH in LMICs lack access to cervical cancer screening. Despite tremendous gains made in access to life prolonging antiretroviral therapy for WLWH, most are served by vertical human immunodeficiency virus (HIV) programmes which do not integrate these two crucial services. We present a case of a WLWH, in HIV care for a decade, who was recently diagnosed with preventable, advanced stage cervical cancer.

## Background

Although cervical cancer is preventable, in 2018, an estimated 570,000 new cases and 311,000 deaths due to cervical cancer occurred globally [1]. The burden of cervical cancer disproportionately affects low- and middle-income countries (LMICs), which accounted for 90% of deaths in 2018 [2]. Women living with Human Immunodeficiency Virus (WLWH), the majority of whom reside in LMICs, are at increased risk of cervical cancer due to higher prevalence and persistence of human papillomavirus (HPV), the causative agent [3]. Compared to human immunodeficiency virus (HIV)-negative women, WLWH have a shorter time from HPV infection to development of precancerous lesions [4], and have a six to eightfold increased risk of developing invasive cervical cancer [5, 6], making prevention efforts an urgent priority among WLWH.

Effective cervical cancer screening and prevention (CCSP) methods exist. In the USA, the incidence of cervical cancer has decreased by 70% over the last 60 years following introduction of cytology-based screening, and more recently, co-testing for HPV [7]. LMICs have been unable to implement cytology-based screening due to infrastructure requirements, including laboratories and pathologists, and the need for multiple visits [2]. In 2013, the World Health Organization (WHO) recommended cervical cancer screening using Visual Inspection with Acetic Acid (VIA) or HPV testing in LMICs, followed by immediate treatment without histopathologic verification [2]. This ‘screen & treat’ approach, which includes WLWH, is associated with a reduction in high-grade cervical dysplasia and mortality from cervical cancer [2]. Screening and treatment of cervical pre-cancerous lesions is considered a ‘best buy’ for prevention and control of non-communicable diseases by the WHO [1], which recently launched a strategy for global elimination of cervical cancer.

Despite evidence-based guidelines, access to routine CCSP in LMICs is limited, explaining the high mortality burden. A 2015 Demographic and Health Survey in Kenya found that only 16.4% of women aged 30–49 years had ever undergone cervical cancer screening [8]. WLWH in LMICs, despite their higher risk of invasive cancer, similarly lack access to routine screening and prevention [5]. With increased access to antiretroviral therapy (ART), and declining HIV-related mortality, cervical cancer is becoming a leading cause of death for the 16 million WLWH [5], diminishing hard-fought gains. Access to CCSP is especially critical as the incidence of cervical cancer has not decreased despite increased access to ART[5].

In Western Kenya, where we work as part of Family AIDS Care & Education Services (FACES), a Centers for Disease Control/President's Emergency Plan for AIDS Relief (PEPFAR)-supported implementing partners in Kisumu County, Kenya [9], the HIV prevalence is 18%, approximately three times the national average [10]. Similar to many LMICs, access to cervical cancer screening for WLWH in Kenya is sporadic, and often limited to vertical programmes with limited time frame and funding. The lack of routine, integrated and accountable CCSP services within HIV clinics exposes WLWH to avoidable morbidity and mortality from this preventable cancer. To illustrate the harmful effects of vertical and non-integrated programming, we present a case of a WLWH, enrolled in HIV care for almost a decade, recently diagnosed with invasive cervical cancer at a FACES-supported Ministry of Health (MoH) facility.

### Patient PAO

PAO is a 39-year-old Gravida 4 Para 4 diagnosed with HIV on 18 January 2010. PAO is Muslim, married with an 8th-grade education and is unemployed. She enrolled in HIV care on 22 January 2010, with an initial CD4 cell count of 27 cells/mm^3^ and has remained at the same facility throughout. She started on ART at enrolment and has visited the HIV clinic on average every 3 months since enrolment. Similar to many HIV clinics in Kenya, PAO’s HIV clinic does not offer cervical cancer screening, and instead relies on neighbouring government clinics to offer this service. In October of 2011, approximately 22 months after enrolling in HIV care, PAO had VIA screening at the HIV clinic for the first time as part of a research study. She was VIA-positive, and a biopsy was taken. The study was transitioned to the MoH soon after, and there are no records of her biopsy results or any follow-up treatment. Following the transition of the CCSP services to the MoH, PAO's HIV clinic did not continue to offer routine CCSP services due to a weak supply chain and competing priorities amidst a stretched workforce focused strictly on HIV-specific targets. She did not undergo further cervical cancer screening until 6 January 2020, when a different research study began to offer screening via HPV self-sampling at her clinic. At presentation for self-testing in 2020, PAO, whose CD4 had since risen to 463 cells/mm^3^ was ill-appearing, and endorsed blood-tinged vaginal discharge and generalised malaise for 2 months. A speculum exam revealed a fungating necrotic mass with friable tissue occupying the posterior cervix. Clinical suspicion of cervical cancer was made, and a biopsy was obtained. Following the biopsy, PAO experienced copious vaginal bleeding, with a point of care haemoglobin of 6.0 mg/dL. The vagina was packed, and she was transferred to the local referral hospital. At the hospital, PAO waited 1 week to obtain a blood transfusion due to a lack of blood at the regional blood bank and was discharged home following transfusion. Biopsy results were available 3 weeks later, revealing moderately differentiated squamous cell carcinoma of the cervix. The sole gynaecologic oncologist in the region saw PAO on 3 March 2020, approximately 8 weeks following her initial presentation. On examination, she received a Stage 2B cervical cancer diagnosis, and was recommended neoadjuvant chemotherapy while awaiting national health insurance approval for radiation therapy, which PAO could afford to pay for out of pocket. Initiation of neoadjuvant chemotherapy was further delayed by the need for an additional blood transfusion and baseline labs, which were delayed due to a lack of reagents at the referral hospital laboratory. PAO received her first neoadjuvant chemotherapy on 16th March 2020, approximately 3 weeks after pathology diagnosis of cervical cancer. Her National Health Insurance Fund matured on 11th April 2020. PAO received approximately 28 rounds of radiation therapy but unfortunately died of complications of radiation in January 2021, approximately 1 year after diagnosis.

## Discussion

Significant gains have been made in recent years to improve access to ART for people living with HIV. These gains have led to HIV becoming a chronic disease in many parts of the world. For WLWH in LMICs, these gains are at risk of being lost due to the lack of routine, integrated cervical cancer screening within HIV clinics, as is illustrated by PAO’s case. The WHO recommends cervical cancer screening for HIV positive women at the time of HIV diagnosis, regardless of age, with rescreening every 3 years following a negative result [2]. The Kenya National Cancer Guidelines recommend annual cervical cancer screening for HIV positive women [11]. Despite this, access to CCSP for WLWH in LMICs is limited and often unavailable in HIV clinics where the majority of WLWH receive care. It is not surprising then that given the elevated risk among WLWH, cervical cancer has become a leading cause of death in this population [5]. Weak health systems and limited access to cancer treatment in LMICs present a double burden for WLWH diagnosed with cervical cancer. Survival statistics following cervical cancer diagnosis among WLWH in LMICs are grim, with a Ugandan study finding only a 30% survival rate at 24 months, despite ART and radiation access [12].

Improving outcomes for WLWH requires the integration of cervical cancer screening into routine HIV care. The majority of the global HIV response are vertical programmes, focusing only on HIV care, ART access and viral suppression, despite CCSP being a crucial aspect of care for WLWH. For example, PEPFAR, which funds a majority of HIV care in LMICs, has not traditionally funded CCSP services. This leaves CCSP services to Ministries of Health, where low investments in health, lack of prioritisation and competing demands often means lack of cervical cancer prevention services. This was the case for PAO, illustrating the human cost of this disconnect in funding streams and prioritisation, and calls for urgent integration of cervical cancer screening into all routine HIV care.

Integration of CCSP activities within all HIV clinics through multilateral support including funding from ministries of health, international HIV funding programmes like PEPFAR and local partners is a moral imperative if we are to sustain the gains made in the fight against HIV for women in LMICs. PAO’s case also illustrates the critical need for strengthening referral pathways to enable timely access to pathology diagnoses and cancer treatment following diagnosis. Though PAO had a cervical biopsy performed years prior to her cervical cancer diagnosis, gaps in care between her HIV clinic and pathology services resulted in lack of appropriate follow-up. As such, integration of CCSP initiatives within HIV-clinics must include linkage to pathology and referral services for impact. Furthermore, following the diagnosis of cervical cancer, PAO experienced delays in initiation of life prolonging therapy due to cost and infrastructure barriers at the referral hospital, calling for strengthening of tertiary institutions in LMICs to care for women diagnosed with cancer.

## Conclusion

Despite significant gains in the fight against HIV/AIDS in LMICs resulting in millions of HIV-positive women with prolonged life-expectancy, lack of integration of cervical cancer screening and pre-cancer treatment services in HIV clinics is resulting in preventable mortality and lost gains in this high-risk population.

## Conflict of interest

The authors have no conflict of interest to declare.

## Author’s contribution

All authors contributed equally to this manuscript.
